# Supplementary material for: Discovery of a Therapeutic Agent for Glioblastoma Using a Systems Biology-Based Drug Repositioning Approach
Source: Int J Mol Sci. 2024 Jul 18;25(14):7868. doi: 10.3390/ijms25147868 (PMC11277330; doi:10.3390/ijms25147868)
Supplement: Supplementary file 1 [file ijms-25-07868-s001.zip › Supplementary File SA.pdf]

Supplementary information

Discovery of a therapeutic agent for Glioblastoma using a systems biology-based drug repositioning approach

Ali Kaynar, Mehmet Ozcan, Xiangyu Li, Hasan Turkez, Cheng Zhang, Mathias Uhlén, Saeed Shoaie and Adil Mardinoglu

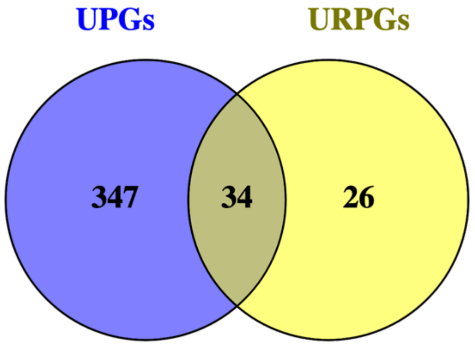

UPGs	URPGs	Overlaps	P value
381	60	34	0

Figure S1. Significant Overlap of UPGs and URPGs Indicated by Hypergeometric Test, related to DEG Analysis Supports Survival Results. Comparison of the Cox Unfavourable gene pool (UPGs) and DGE upregulated genes (URPGs) via hypergeometric test indicates significant overlaps.

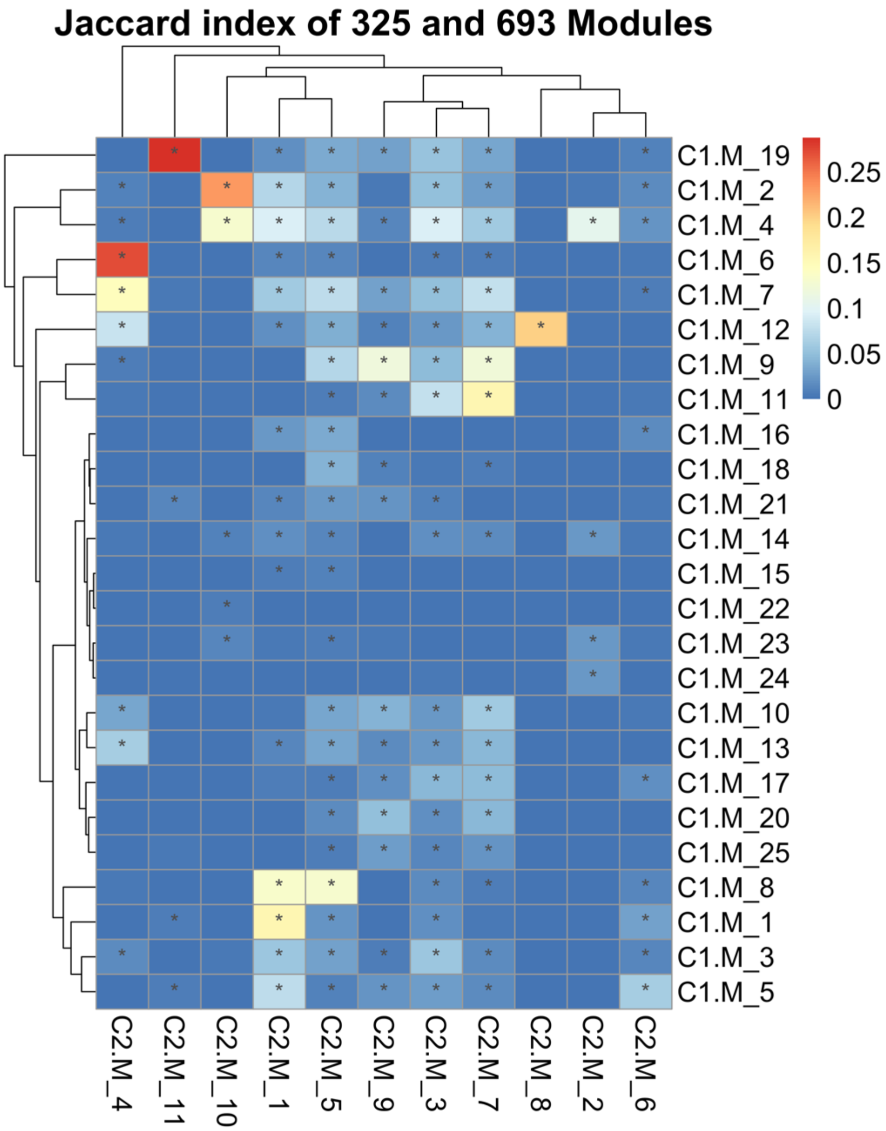

Figure S2. Comparison of modules from CGGA_325 and CGGA_693 based on the hypergeometric test, related to WGCNA Analysis. All possible significant overlap module pairs are shown. For the module name, the C1 suffix stands for CGGA_325, and C2 stand for CGGA_693. *P value < 0.05

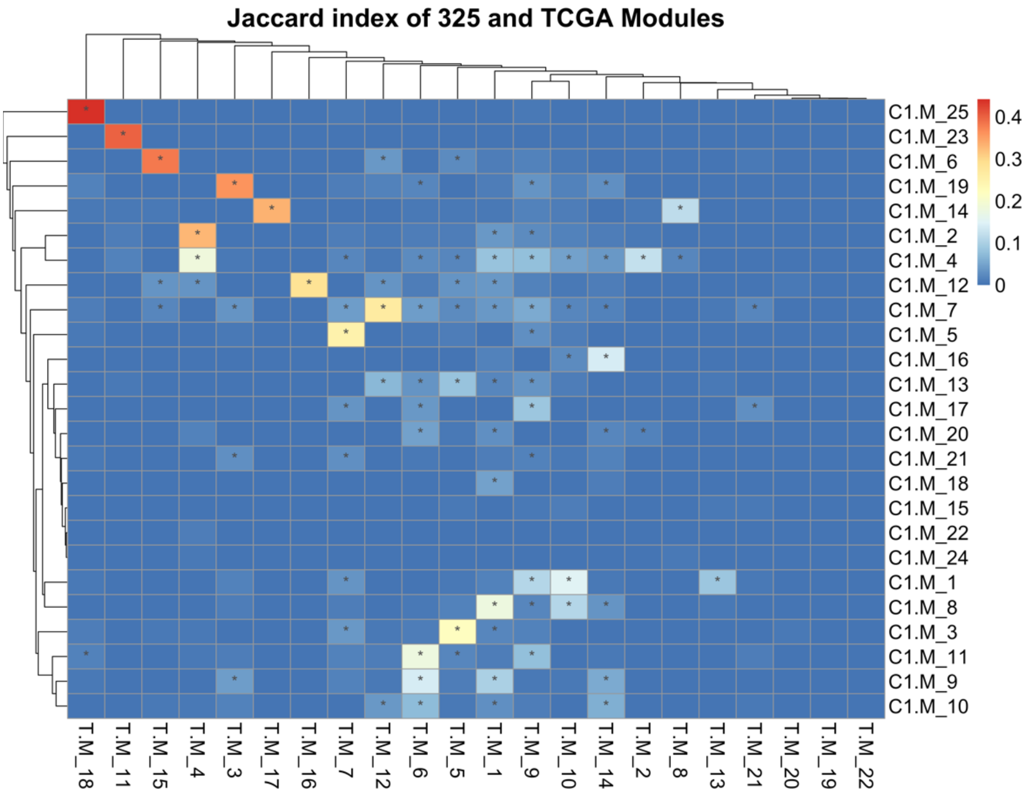

Figure S3. Comparison of modules from CGGA_325 and TCGA based on the hypergeometric test, related to WGCNA Analysis. All possible significant overlap module pairs are shown. For the module name, the C1 suffix stands for CGGA_325, and T stand for TCGA. *P value < 0.05

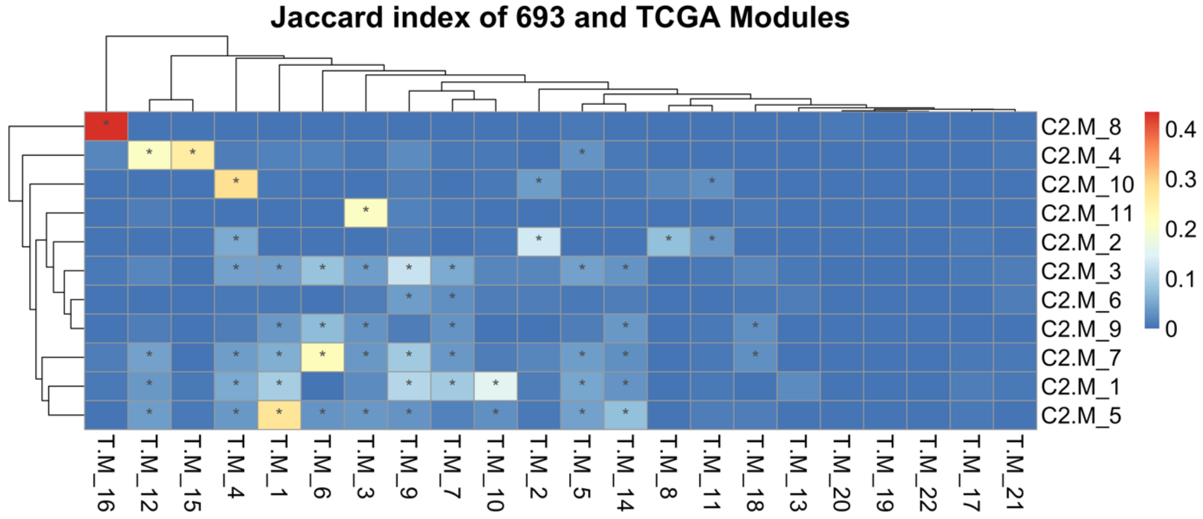

Figure S4. Comparison of modules from CGGA_693 and TCGA based on the hypergeometric test, related to WGCNA Analysis. All possible significant overlap module pairs are shown. For the module name, the C2 suffix stands for CGGA_693, and T stand for TCGA. *P value < 0.05

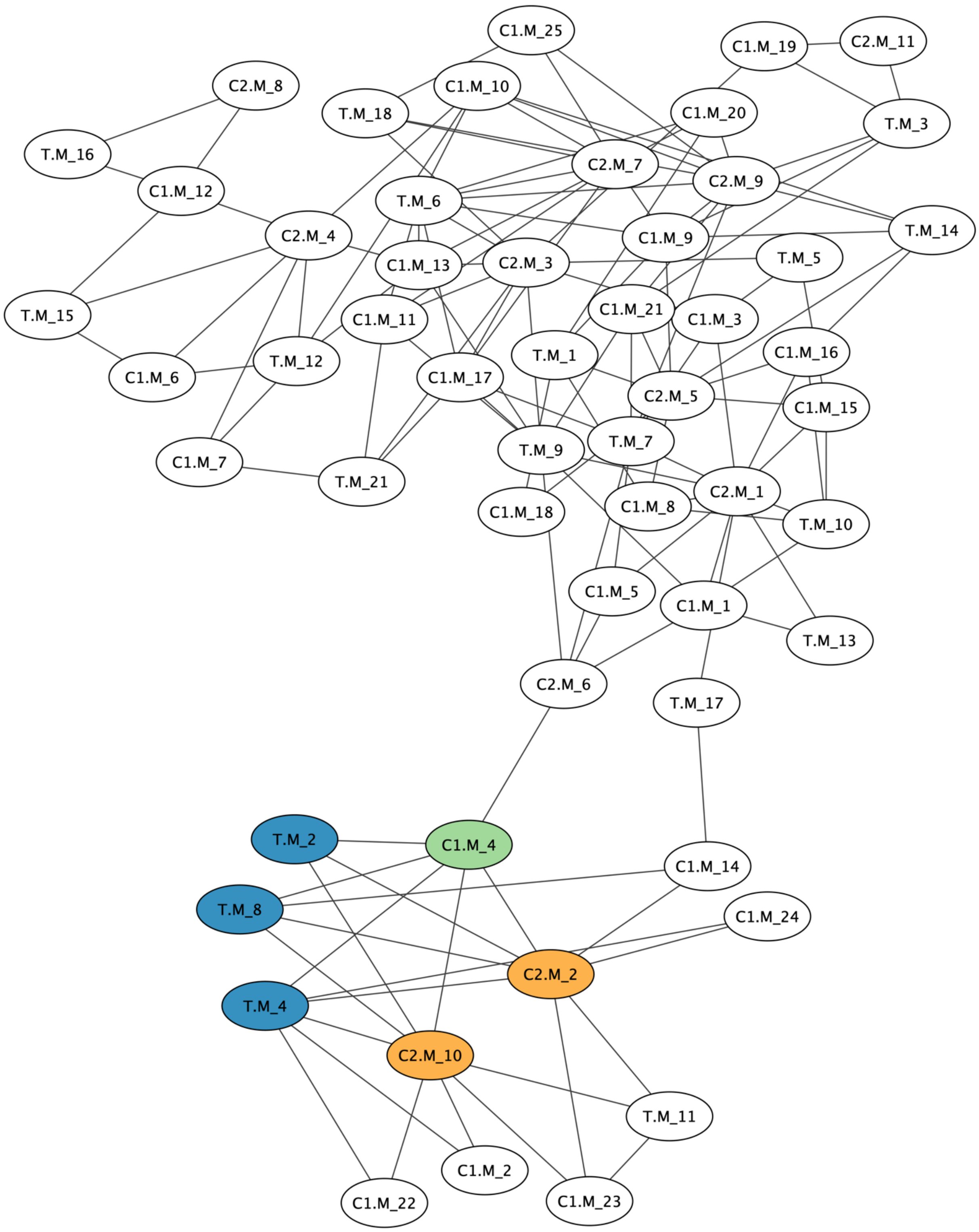

Figure S5. Network of Significantly Overlapped Modules, related to WGCNA modules and UPGs. Each edge represents a significant overlap between each module pair. Blue, orange, and green coloured modules significantly overlap with the UPGs. C1 suffix stands for CGGA_325, C2 suffix stands for CGGA_693, and T stand for TCGA. Hypergeometric test p-value ≤ 0.05. The network is generated by using Cytoscape tool.

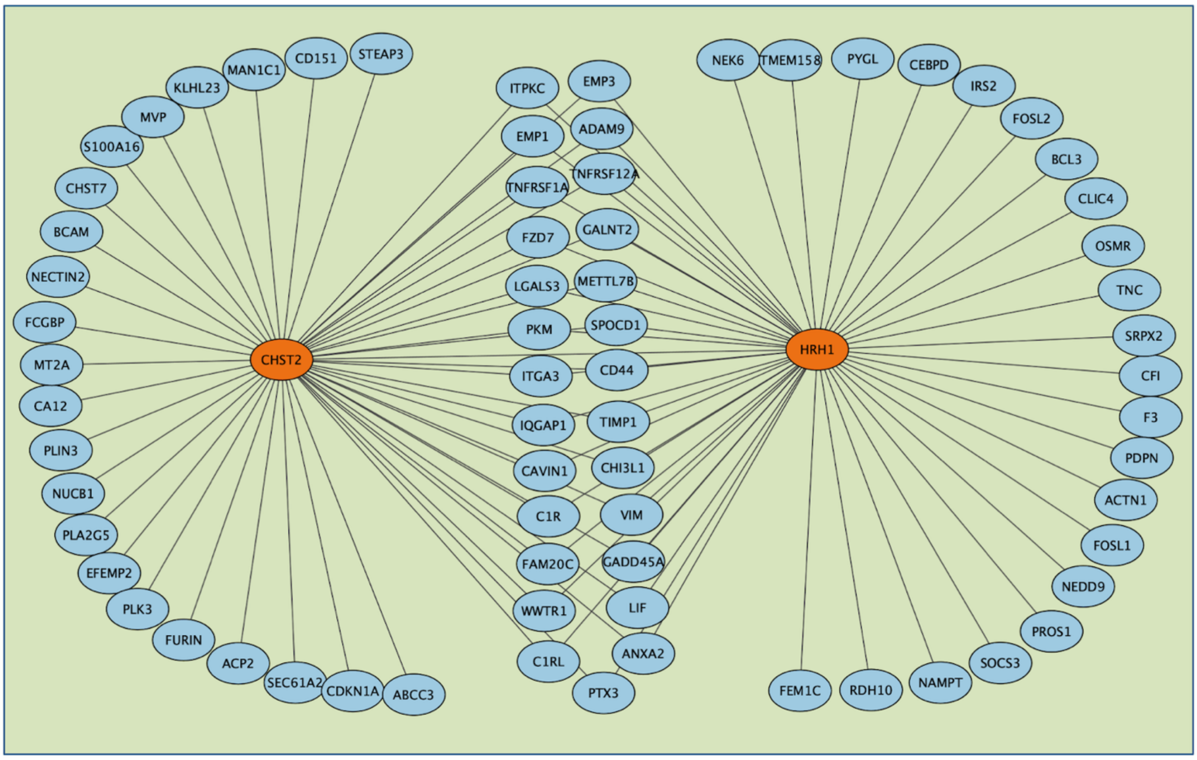

Figure S6: CHST2-HRH1 Co-Expression Network Highlighting Connectivity for Drug Repositioning. Co-expression interaction between CHST2 and HRH1 from iNetModels with 50 node limits. HRH1 is not found in healthy brain cortex data in iNetModels.

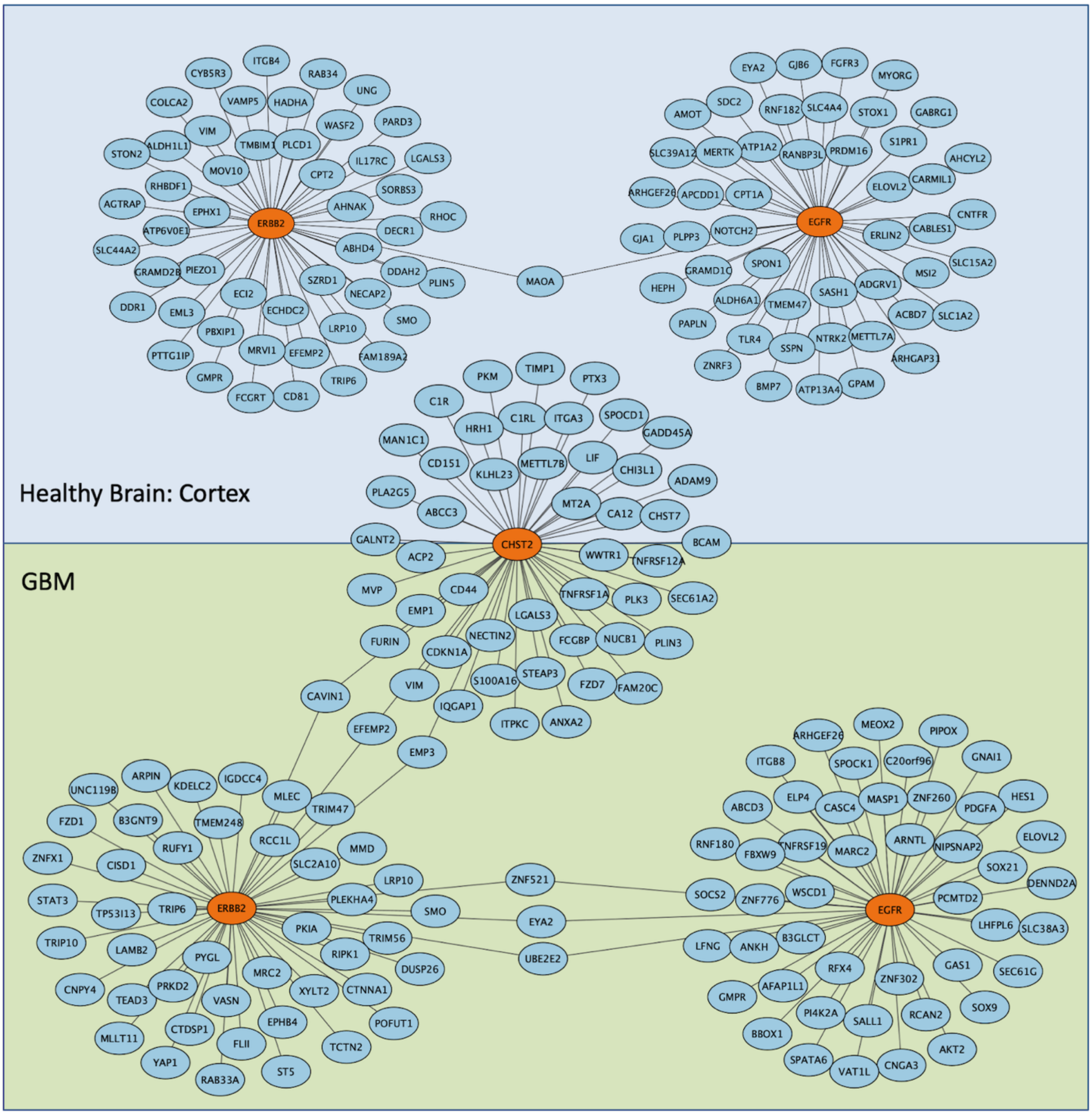

Figure S7: CHST2- ERBB2/EGFR Co-Expression Network Highlighting Connectivity in GBM for Drug Repositioning. Co-expression interaction between CHST2 and ERBB2/EGFR from iNetModels with 50 node limits. There is no connection in the healthy brain cortex region. On the other hand, interactions in the GBM condition provide significant relation between CHST2 and EGFR/ERBB2.

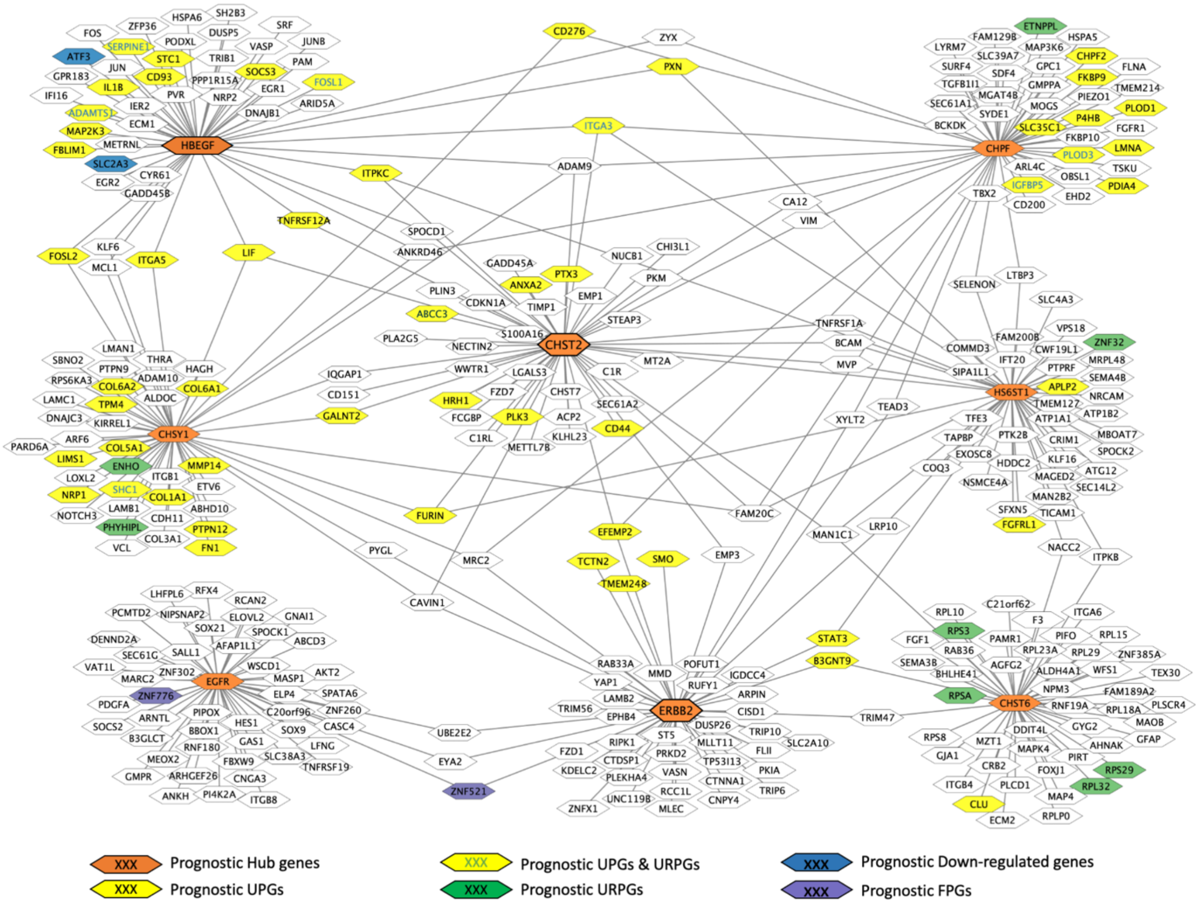

Figure S8. CHST2-Centric Co-expression Map with Significantly Altered GAG Pathway Genes, related to Drug Repositioning. Co-expression network of significantly altered sulfotransferase CHST2, well-known target genes of WZ-4002 drug and some significantly altered genes that have roles in GAG metabolism. Co-expression network created from iNetModels with 50 node limits.

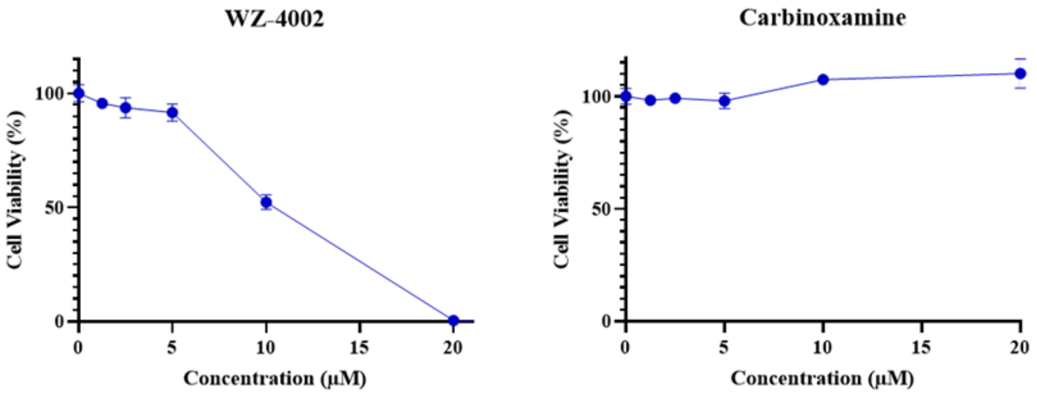

Figure S9. The impact of different concentrations of WZ-4002 and carbinoxamine on cell viability

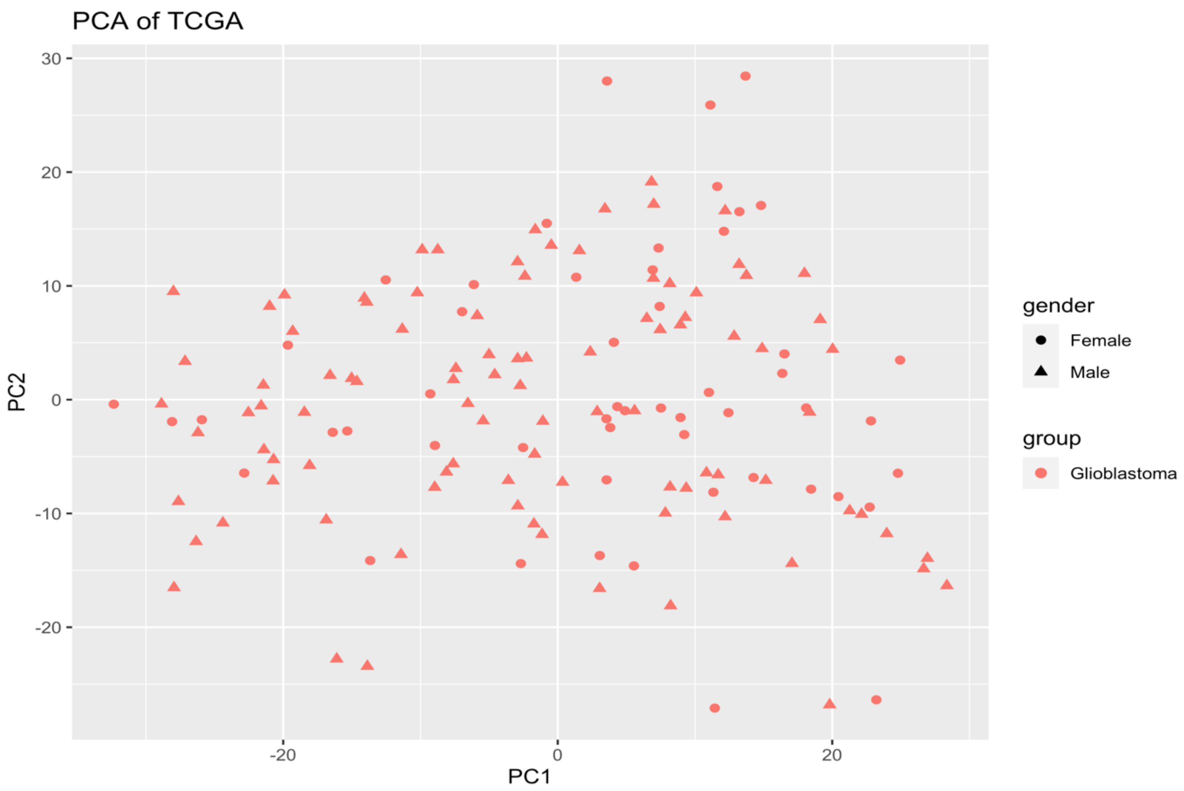

Figure S10: PCA Analysis Confirming Absence of Outliers for TCGA Dataset.

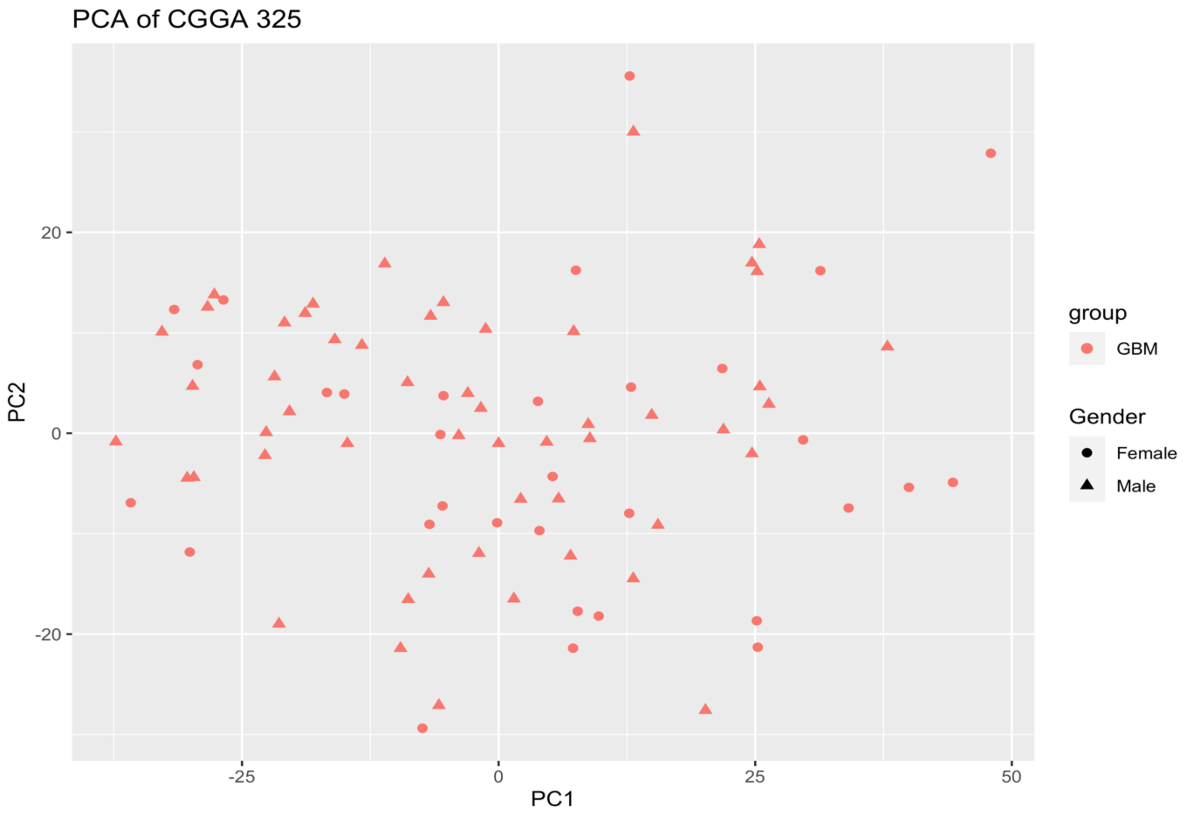

Figure S11: PCA Analysis Confirming Absence of Outliers for CGGA_325.

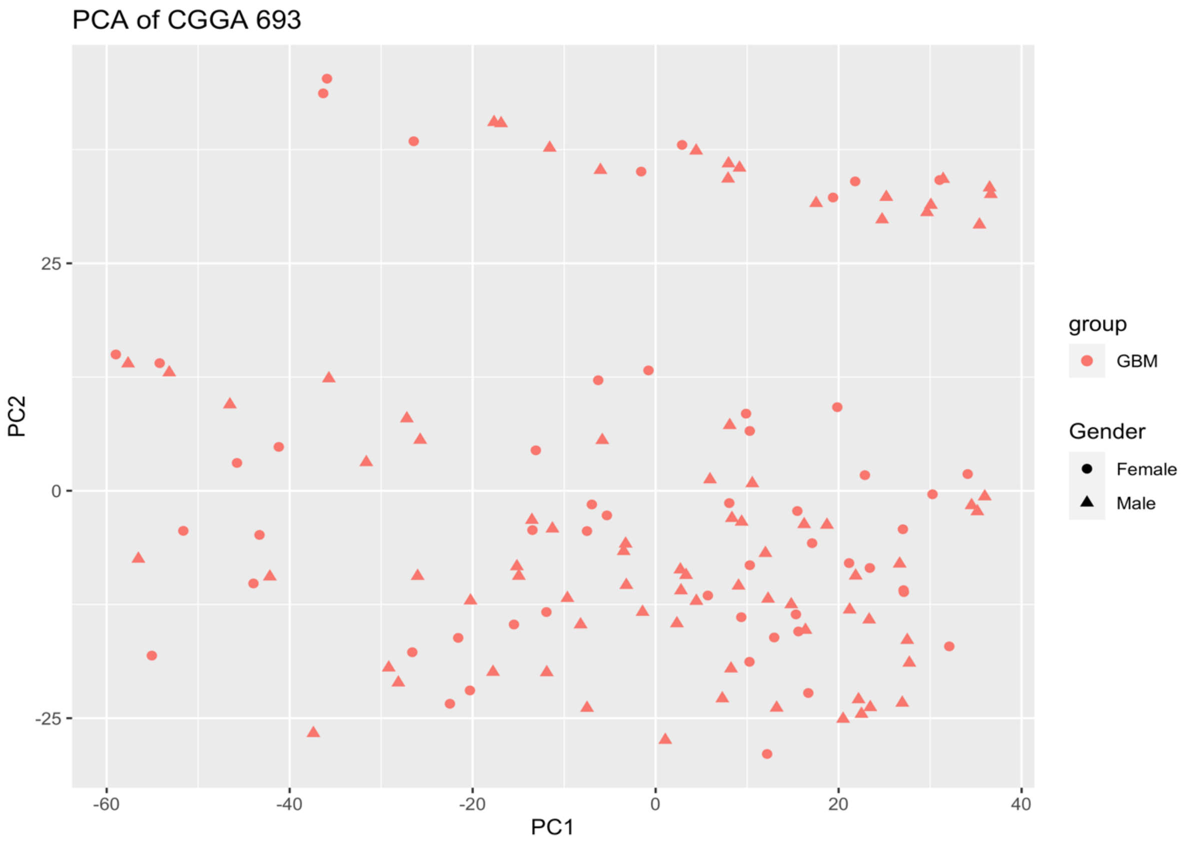

Figure S12: PCA Analysis Confirming Absence of Outliers for CGGA_693.

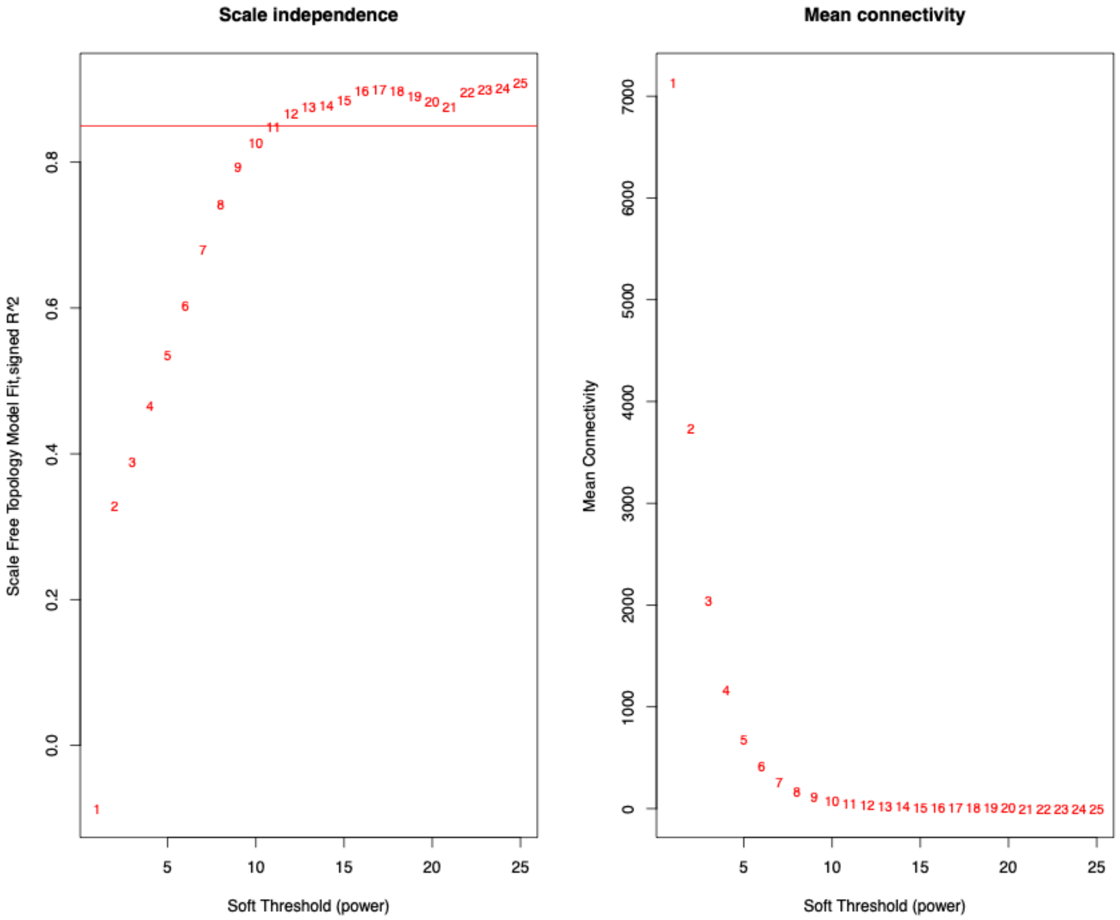

Figure S13: Soft Threshold Optimization for WGCNA in TCGA dataset. R^2 threshold was selected as 0.85. The power parameter was set to 12.

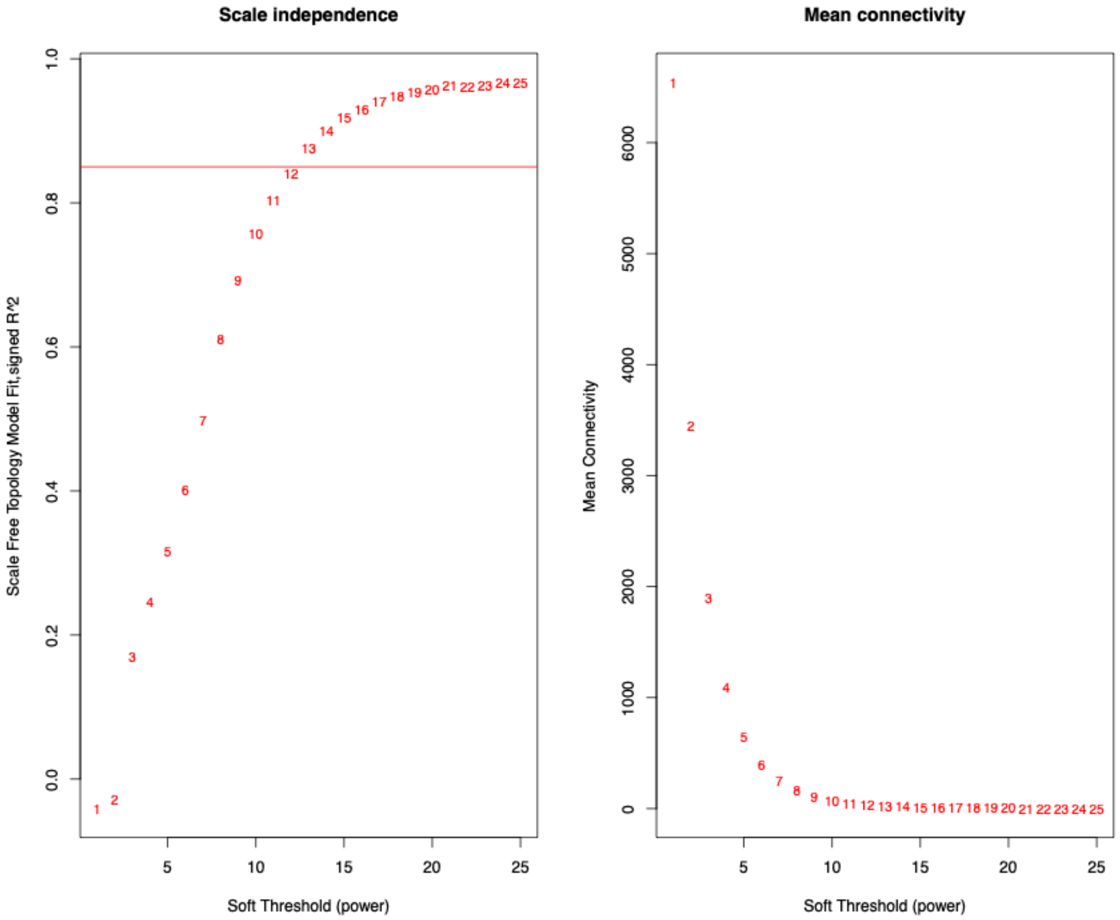

Figure S14: Soft Threshold Optimization for WGCNA in CGGA_325 dataset. R^2 threshold was selected as 0.85. The power parameter was set to 13.

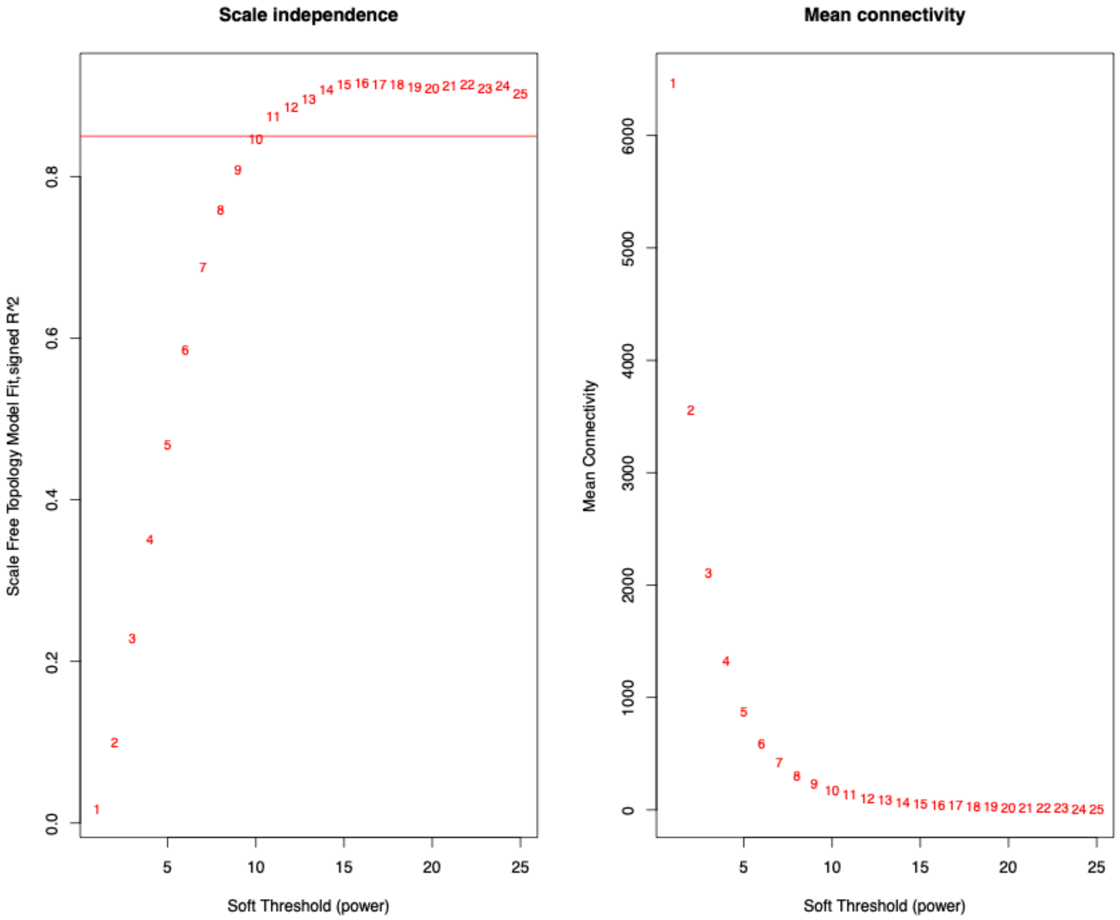

Figure S15: Soft Threshold Optimization for WGCNA in CGGA_693 dataset. R^2 threshold was selected as 0.85. The power parameter was set to 11.

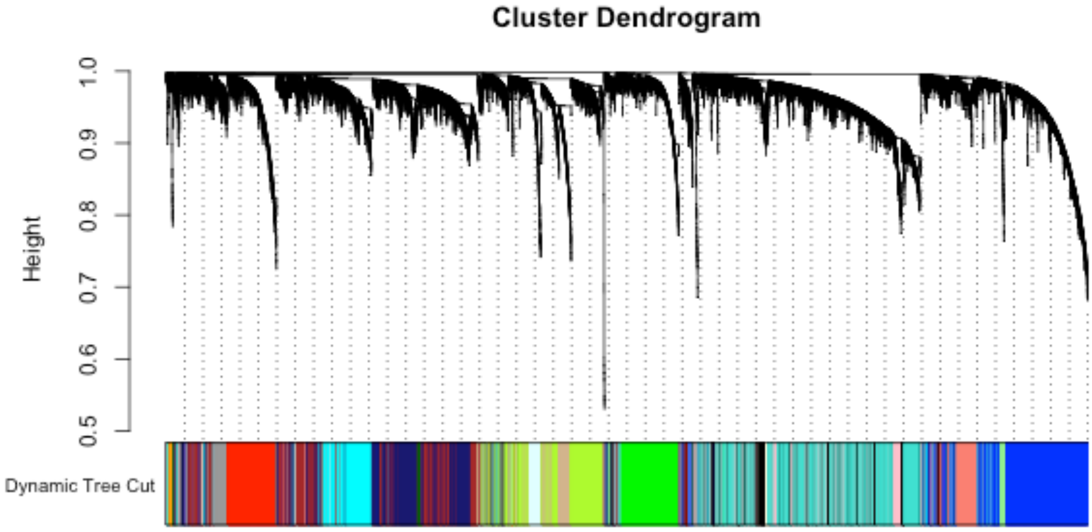

Figure S16: Selection of Final Modules for TCGA Dataset with Minimum Size Threshold of 20

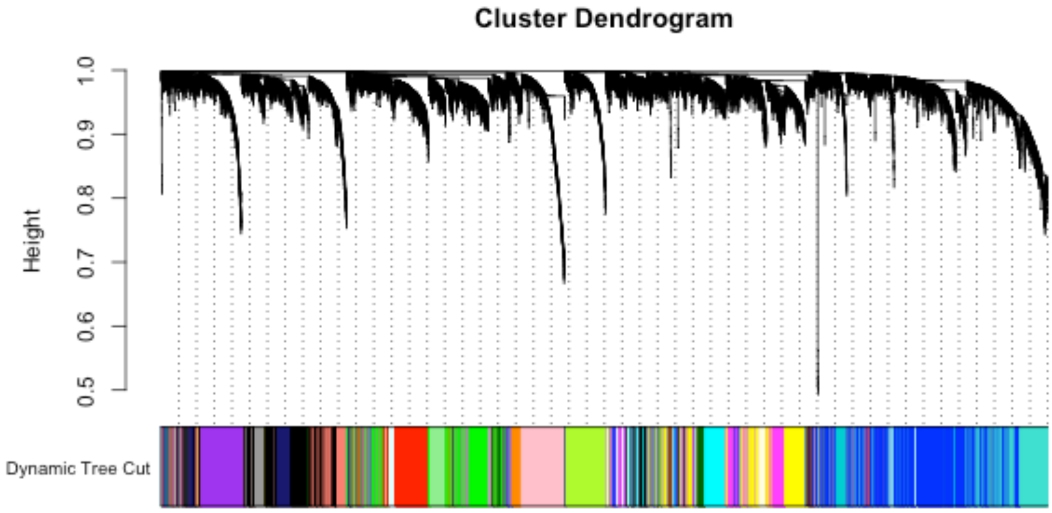

Figure S17: Selection of Final Modules for CGGA_325 Dataset with Minimum Size Threshold of 20.

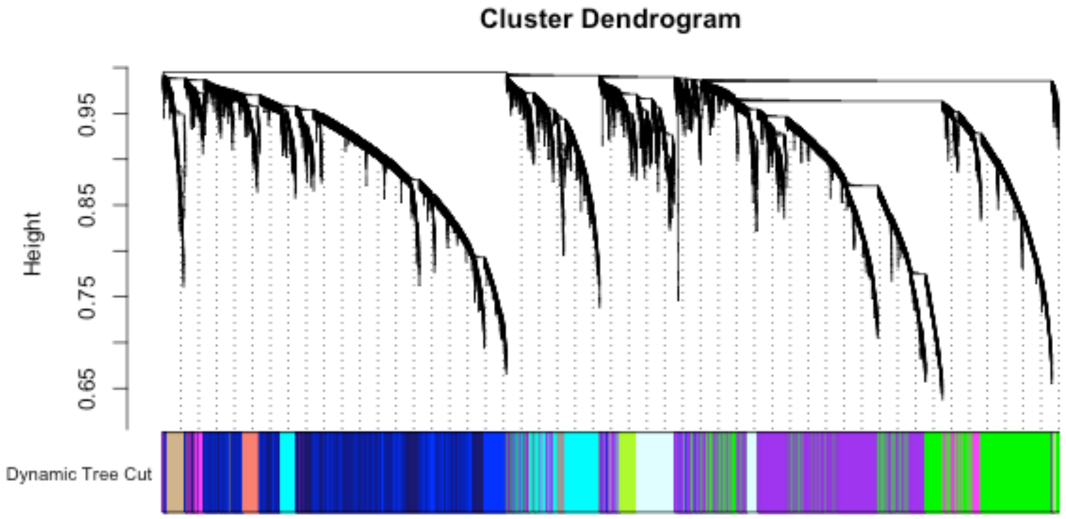

Figure S18: Selection of Final Modules for CGGA_639 Dataset with Minimum Size Threshold of 20

Table S1: Top 25 drugs obtained from the T-Drug database for inhibition of target gene. Displaying the top 25 inhibitory drugs from the T-Drug database, this figure ranks them by their confidence score which signifies the predicted effectiveness in target gene inhibition.

Drug	Target	Effect Type	Num Cells	Num Hit Cells	Hit Rate (%)	Hit Cells	Confidence Score	Well-known target
estradiol (BRD-K18910433)	CHST2	Inhibition	4	2	50	MCF7/A549	0.5437	
importazole (BRD-A02481876)	CHST2	Inhibition	4	2	50	MCF7/A549	0.5207	
barasertib-HQPA (BRD-K63923597)	CHST2	Inhibition	4	2	50	MCF7/A549	0.5204	AURKB
curcumin (BRD-K07572174)	CHST2	Inhibition	4	2	50	A549/MCF7	0.5194	
BRD-A11087911 (BRD-A11087911)	CHST2	Inhibition	4	2	50	A549/MCF7	0.518	
retinol (BRD-K22429181)	CHST2	Inhibition	4	2	50	A549/MCF7	0.5169	RXRB
BRD-K14790374 (BRD-K14790374)	CHST2	Inhibition	4	2	50	MCF7/A549	0.516	
BRD-K86693415 (BRD-K86693415)	CHST2	Inhibition	4	2	50	A549/MCF7	0.5152	
BRD-K09907482 (BRD-K09907482)	CHST2	Inhibition	4	2	50	MCF7/A549	0.5147	
SB-216763 (BRD-K59184148)	CHST2	Inhibition	4	2	50	A549/MCF7	0.5141	GSK-3β
**carbinoxamine (BRD-A29426959)**	**CHST2**	**Inhibition**	**4**	**2**	**50**	**MCF7/A549**	**0.5108**	**HRH1**
PF-562271 (BRD-K99545815)	CHST2	Inhibition	4	2	50	A549/MCF7	0.5083	
ropinirole (BRD-K15933101)	CHST2	Inhibition	4	2	50	A549/MCF7	0.5042	
desoxypeganine (BRD-K13819402)	CHST2	Inhibition	4	2	50	MCF7/A549	0.5033	
BRD-K06083657 (BRD-K06083657)	CHST2	Inhibition	4	2	50	A549/MCF7	0.5026	
PT-630 (BRD-A73680854)	CHST2	Inhibition	4	2	50	MCF7/A549	0.5009	
CD-00491SC (BRD-K99311057)	CHST2	Inhibition	4	2	50	A549/MCF7	0.5001	
brivudine (BRD-A63675168)	CHST2	Inhibition	4	2	50	A549/MCF7	0.4973	
ticlopidine (BRD-K00603606)	CHST2	Inhibition	4	2	50	A549/MCF7	0.4966	
JNJ-38877605 (BRD-K02123250)	CHST2	Inhibition	4	2	50	MCF7/A549	0.495	
tamoxifen (BRD-K93754473)	CHST2	Inhibition	4	2	50	MCF7/A549	0.4942	
SID-26681509 (BRD-K08417745)	CHST2	Inhibition	4	2	50	MCF7/A549	0.4912	
**WZ-4002 (BRD-K72420232)**	**CHST2**	**Inhibition**	**4**	**2**	**50**	**A549/MCF7**	**0.489**	**EGFR/ERBB2**
BRD-K02969269 (BRD-K02969269)	CHST2	Inhibition	4	2	50	A549/MCF7	0.4885	
kinetin-riboside (BRD-K94325918)	CHST2	Inhibition	4	2	50	MCF7/A549	0.4882	

Table S2: Drug-Target Pairs and Activity Types Derived from CMAP. This table lists drugs from Table S1 and their well-known target genes from the CMAP database, including the drug's activity type.

Drug ID	Drug Name	Well-known target	Activity
BRD-K63923597	barasertib-HQPA	AURKB	Aurora kinase inhibitor
**BRD-K72420232**	**WZ-4002**	**EGFR**	**EGFR inhibitor**
**BRD-K72420232**	**WZ-4002**	**ERBB2**	**EGFR inhibitor**
BRD-K59184148	SB-216763	GSK3B	GSK-3 inhibitor
BRD-K08417745	SID-26681509	CTSL	Cathepsin inhibitor
BRD-K07572174	curcumin	PTGS1	Lipoxygenase inhibitor
BRD-K07572174	curcumin	PTGS2	Lipoxygenase inhibitor
BRD-K07572174	curcumin	PTGS1	NFKB pathway inhibitor
BRD-K07572174	curcumin	PTGS2	NFKB pathway inhibitor
BRD-K07572174	curcumin	PTGS1	Cyclooxygenase inhibitor
BRD-K07572174	curcumin	PTGS2	Cyclooxygenase inhibitor
BRD-K22429181	retinol	RXRA	Retinoid receptor ligand
BRD-K22429181	retinol	RXRB	Retinoid receptor ligand
BRD-K22429181	retinol	RXRG	Retinoid receptor ligand
BRD-K15933101	ropinirole	DRD2	Dopamine receptor agonist
BRD-K15933101	ropinirole	DRD3	Dopamine receptor agonist
BRD-K18910433	estradiol	ESR1	Estrogen receptor agonist
BRD-K18910433	estradiol	ESR2	Estrogen receptor agonist
BRD-K18910433	estradiol	NR1I2	Estrogen receptor agonist
BRD-K02123250	JNJ-38877605	MET	Tyrosine kinase inhibitor
BRD-K13819402	desoxypeganine	MAOA	Monoamine oxidase inhibitor
BRD-K13819402	desoxypeganine	ACHE	Monoamine oxidase inhibitor
BRD-K93754473	tamoxifen	ESR1	Estrogen receptor antagonist
BRD-K93754473	tamoxifen	ESR2	Estrogen receptor antagonist
BRD-A29426959	carbinoxamine	HRH1	Histamine receptor antagonist
BRD-K13819402	desoxypeganine	MAOA	Acetylcholinesterase inhibitor
BRD-K13819402	desoxypeganine	ACHE	Acetylcholinesterase inhibitor
BRD-A73680854	PT-630	DPP4	Dipeptidyl peptidase inhibitor
**BRD-A29426959**	**carbinoxamine**	**HRH1**	**L-type calcium channel blocker**
BRD-K00603606	ticlopidine	P2RY12	Purinergic receptor antagonist
BRD-K99545815	PF-562271	PTK2B	Focal adhesion kinase inhibitor
BRD-K99545815	PF-562271	PTK2	Focal adhesion kinase inhibitor
BRD-K07572174	curcumin	PTGS1	Histone acetyltransferase inhibitor
BRD-K07572174	curcumin	PTGS2	Histone acetyltransferase inhibitor
BRD-K93754473	tamoxifen	ESR1	Selective estrogen receptor modulator
BRD-K93754473	tamoxifen	ESR2	Selective estrogen receptor modulator
BRD-A02481876	importazole	KPNB1	Importin-beta transport receptor inhibitor

Table S3: Overview of Candidate Genes and their RNAi and CRISPR Screening Results (Extended version of Table 1). Information about candidate Target genes. In the DeepMap Graphic, the graph colour represents RNAi (Achilles+DRIVE+Marcotte, DEMETER2), Blue colour represents CRISPR (DepMap Public 22Q4+Score, Chronos). *Elevated in brain tissue or cerebrospinal fluid, ** Brain-specific.

Genes	***DepMap Graphic***	***Short Information***
ARRDC4 *	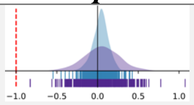	Arrestin domain containing 4 is involved in the regulation of cell growth and survival.
CHST2 *	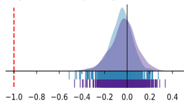	Carbohydrate sulfotransferase 2 is involved in the synthesis of sulfated proteoglycans and plays a role in the extracellular matrix.
CHST6 *	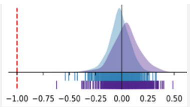	Carbohydrate sulfotransferase 6 is involved in the synthesis of sulfated proteoglycans and plays a role in the extracellular matrix.
CLU *	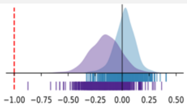	Clusterin is involved in the extracellular matrix and it is important for cell adhesion and migration.
DIRAS3 *	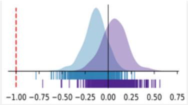	DIRAS family GTPase 3 is involved in the regulation of cell growth and survival.
EN1 *	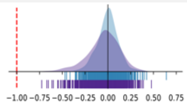	Engrailed homeobox 1 is involved in the development of the nervous system and plays a role in axon guidance.
GLIS3 *	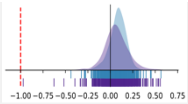	GLIS family zinc finger 3 is involved in the regulation of gene expression and plays a role in the development of the kidney.
GNA12 *	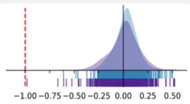	G protein subunit alpha 12 is involved in the regulation of cell growth and survival.
IBSP *	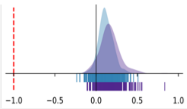	Integrin-binding sialoprotein is involved in the extracellular matrix and is important for cell adhesion and migration.
LCTL *	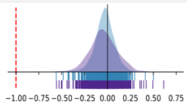	Lactase-like, the function of which is to hydrolyse glycosidic bonds and involved in sensory transduction.
LZTS1 *	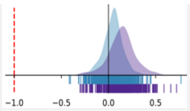	Leucine zipper, putative tumour suppressor 1 is involved in the regulation of cell growth and survival.
MT1F *	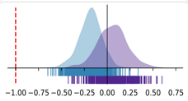	Metallothionein 1F is involved in the regulation of metal ions and plays a role in the response to oxidative stress.
SCARA3 *	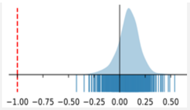	Scavenger receptor class A member 3 is involved in the recognition and clearance of damaged cells and plays a role in the immune system.
DRAXIN **	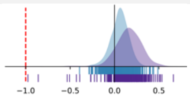	Dorsal inhibitory axon guidance protein is involved in the development of the nervous system and plays a role in axon guidance.
